# Effect of Benson's relaxation technique on caregiver burden in caregivers of hemodialysis patients. A Randomized Controlled Trial*

**DOI:** 10.17533/udea.iee.v40n3e06

**Published:** 2023-02-10

**Authors:** Mahsa Imanian, Somayeh Ramezanli

**Affiliations:** 1 Nurse Instructor, M.Sc. Department of Nursing, Jahrom University of Medical Sciences, Jahrom, Iran. Email: mahsa.imanian@gmail.com Jahrom University Department of Nursing Jahrom University of Medical Sciences Jahrom Iran mahsa.imanian@gmail.com; 2 Nurse Instructor, M.Sc. Department of Nursing, Jahrom University of Medical Sciences, Jahrom, Iran. Email: ramezanli1362@gmail.com. Corresponding author. Jahrom University Department of Nursing Jahrom University of Medical Sciences Jahrom Iran ramezanli1362@gmail.com.

**Keywords:** caregiver burden, control groups, relaxation therapy, renal dialysis, kidney failure, chronic., carga del cuidador, grupos control, terapia de relajación, diálisis renal, fallo renal crónico, fardo do cuidador, grupos controle, terapia de relaxamento, diálise renal, falência renal crónica

## Abstract

**Methods.:**

This is a randomized controlled trial study on 52 caregivers of hemodialysis patients referred an universitary hospital at Jahrom. The caregivers were randomly divided into intervention and control groups. In the intervention group, Benson's relaxation was performed twice a day for 15 minutes each time, and continued for one month. Data collection tools included demographic information questionnaire and standard Zarit Burden Interview questionnaire which was completed by all participants before the intervention and one month after it.

**Results.:**

After the intervention, the mean caregiver burden of hemodialysis patients in the intervention group decreased significantly compared to the control group (*p*<0.001). The results of paired t-test showed that in the intervention group, the mean scores of caregiver burden after the intervention (14.46± 10.91) was significantly lower than before the intervention (38.33±16.94) (*p*=0.001).

**Conclusion.:**

Benson's relaxation method can reduce caregiver burden in caregivers of hemodialysis patients.

## Resumen

**Objetivo.:**

Investigar el efecto de la técnica de relajación de Benson sobre la carga de los cuidadores de pacientes en hemodiálisis.

**Métodos.:**

Estudio de intervención realizado con la participación de 52 cuidadores de pacientes en hemodiálisis remitidos a un hospital universitario de Jahrom (Iran). Los cuidadores fueron divididos aleatoriamente en los grupos de intervención y de control. En el grupo de intervención, se realizó la técnica de relajación de Benson dos veces al día durante 15 minutos cada vez, y se continuó durante un mes. Las herramientas de recogida de datos incluían un cuestionario de información demográfica y la entrevista de percepción de carga del cuidador “Zarit Burden Interview” que fue completado por todas participantes antes de la intervención y un mes después de terminada la misma.

**Resultados.:**

Después de la intervención, la carga media de los cuidadores de pacientes en hemodiálisis en el grupo de intervención disminuyó significativamente en comparación con el grupo de control (*p*<0.001). Los resultados de la prueba t pareada mostraron que en el grupo de intervención, las puntuaciones medias de la carga del cuidador después de la intervención (14.46±10.91) fueron significativamente menores que antes de la intervención (38.33±16.94) (*p*=0.001).

**Conclusión.:**

El método de relajación de Benson puede reducir la carga de los cuidadores de pacientes en hemodiálisis.

## Resumo

**Objetivo.:**

Investigar o efeito do relaxamento de Benson na sobrecarga do cuidador em cuidadores de pacientes em hemodiálise na cidade de Jahrom.

**Métodos.:**

Este é um estudo controlado randomizado envolvendo 52 cuidadores de pacientes em hemodiálise encaminhados a um hospital afiliado à Jahrom University of Medical Sciences. Os cuidadores foram divididos aleatoriamente em *grupos intervenção* (*n*=24) e controle (*n*=24). No grupo intervenção, os cuidadores realizaram o relaxamento de Benson duas vezes ao dia por 15 minutos cada, e foi continuado por um mês. A coleta de informações incluiu um questionário de informações demográficas e o questionário padrão Zarit que foi preenchido por todos os participantes antes da intervenção e um mês após o seu preenchimento.

**Resultado:**

S. A maioria dos cuidadores nos grupos intervenção (79,2%) e controle (64,3%) eram mulheres. Após a intervenção, a sobrecarga média dos cuidadores de pacientes em hemodiálise no grupo intervenção diminuiu significativamente em relação ao grupo controle (p<0,001). Os resultados do teste t pareado mostraram que, apenas no grupo intervenção, os escores médios de sobrecarga do cuidador após a intervenção (14,46 ±10,91) foram significativamente menores do que antes da intervenção (38,33) ±16,94) com *p* <0,001.

**Conclusão.:**

O método de relaxamento de Benson demonstrou ser eficaz na redução da sobrecarga dos cuidadores de pacientes em hemodiálise.

## Introduction

Chronic kidney disease is a major and growing health concern all around the world. It is estimated that 750 million people worldwide suffer from chronic kidney disease of which about 3 million are on kidney dialysis.(1) Hemodialysis is a treatment that helps to improve the patient's physical condition by preventing the complications caused by uremia.(2) Moreover, hemodialysis reduces patients' energy levels and affects their ability in doing their daily activities, thereby disrupting the daily lives of patients and their caregivers.(1) Chronic diseases affect not only patients but also their caregivers.(3) For their daily lives and visiting hospital, hemodialysis patients need care and assistance of family and others, which places a significant burden on caregivers. The burden imposed by the care process on the family is mainly due to a combination of physical work, emotional stress, social constraints and economic needs while providing care to their patients, which leads to a significant reduction in the quality of life of caregivers and their health status.(2) Caring for patients with maintenance peritoneal dialysis causes physical, psychological and social stress in family caregivers, so that stress levels are affected by the severity of the disease and other demographic factors in patients and their caregivers.(4)

Also the caregiver burden in the caregivers of these patients increased because of mental and physical disorders caused by chronic kidney failure.(5) Hemodialysis caregivers are often family members or friends who support the patient physically, mentally, and socially. According to studies, caregivers of hemodialysis patients experience a high level of caregiver burden.(1) Caregiver burden may bring about feelings of guilt, disappointment, loneliness, depression, anger, stress, and lack of freedom, all of which could result in severe psychological problems.(6) Caregiver burden is a general term used to describe physical and mental problems as well as the financial cost of the care. Caregiver burden is defined as a persistent difficulty, stress, or negative experience resulting from the care provided by patient caregivers. Therefore, it is so important to assess the condition of caregivers and determine their needs.(7)

One of the methods which can soothe a wide range of physical and mental symptoms such as anxiety, depression, stress and pain is Benson's relaxation technique (BRT).(8) Relaxation can reduce tension in the peripheral muscles, reduce physiological problems and disorders and, through reducing anxiety, can increase physical activity and improve the feeling of well-being.(9) The use of complementary and alternative methods has been gradually increasing in recent years. Herbert Benson developed his technique based on the concepts of transcendent meditation. In this method, one becomes passive and allows calmness to develop and progress. Progressive muscle relaxation (PMR) has been developed to reduce stress and anxiety.(10) This cost-effective method does not require special equipment and can be easily used by people.(8) Generally speaking, patient caregivers are often given less attention and the main focus is usually on patients. Frequent hospitalization of patients and disease-related factors can worsen depression and reduce the quality of life of caregivers.(7)

The process of dialysis has a profound effect on the life of both patients and their caregivers. Moreover, most studies have dealt only with patients, and the interaction between the patient and the family (caregivers) has been overlooked by many researchers. On the other hand, there is a close relationship between the health of caregivers and patients and caregivers can play a significant role in the health and improvement of their hemodialysis patients. Studies show that some interventions such as psycho-educational intervention,(11) family intervention programs,(12) psychosocial Support(5) are effective in reducing the caregiver burden in caregivers of hemodialysis patients. Since the learning and teaching Benson’s relaxation method is easy and requires no specific knowledge and skills and can easily be done by the caregivers of hemodialysis patients and considering the important role of caregivers in the process of caring for hemodialysis patients and the limited studies on the effect of BRT on reducing caregiver burden in this group of caregivers, therefore the present study aims to determine the effect of BRT on caregiver burden in caregivers of hemodialysis patients.

## Methods

### Design and participants

This study was a randomized controlled trial study. The caregivers of dialysis patients who had referred to the dialysis ward of Jahrom University of Medical Sciences in Iran from September to December. Inclusion criteria for the caregivers of the patients consisted of willingness to participate in the study, being 18-60-years-old, literacy and having the most contact with and care of the patient. These criteria for the patients included having at least six months’ history of the disease, three 3-4 hour dialysis session per week and having no history of kidney transplantation. The caregivers who have recently been exposed to stressful events such as accidents, deaths of loved ones, financial losses, etc., as well as those with severe mental and physical disorders, or those who were attending psychological training and counseling sessions were excluded from the study. 

According to the results of caregiver burden in previous study,(13) after intervention in control group(42,18 ± 13,73) and intervention group(31,92 ± 13,98),β=0.20, α= 0.05, effect size=0.74, sample size calculated 30 in each group and totally 60 caregivers.




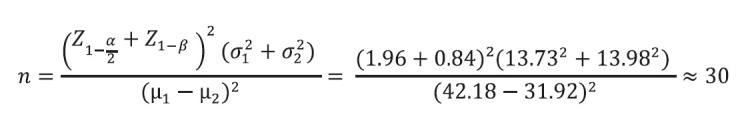




All eligible caregivers of hemodialysis patients were entered to the study by convenience sampling method with random allocation, when the sample size reached 60 sampling was stopped. Then, they were assigned equally into two intervention and control groups by simple randomization, using the table of random numbers and sampling frame (list of caregivers of hemodialysis patients available in the dialysis ward).

Six caregivers of hemodialysis patients in intervention group did not continue the study due to lack of willingness, long distance from their place of residence, failure to complete the BRT and death of patients; also, two caregivers in the control group did not return to the ward to complete the post-test because of the long distance from their place of residence. Eventually, 24 caregivers in the intervention group and 28 in the control group completed the study.

### Data collection tools

Data was collected using questionnaires before and one month after the intervention. The first questionnaire was demographic information questionnaire (e.g. age, sex, education level, marital status, occupation and duration of care). The second instrument was Zarit Burden Interview (ZBI) questionnaire. ZBI-22 was developed by Zarit, Reever and Bach-Peterson in 1980.(14-16) It is the most prevalent assessment tool for measuring the perceived caregiver burden caused by the care provided by the family caregivers. Answers to the questions of the questionnaire are based on a 5-point scale including never (0), rarely (1), sometimes (2), usually (3) and always (4). The sum of the points obtained by the caregivers determines their caregiver burden. A score of less than 20 is rated as mild burden, 21-40 mild to moderate, 41-60 moderate to severe, and 61-88 represents severe burden. The minimum and maximum score of each person is considered to be between zero and 88 and a higher score will indicate higher caregiver burden and vice versa. The reliability coefficient of the original version (0.71) and its internal consistency (0.91) were measured using retest method and Cronbach's alpha.(14) Also in terms of construct validity, the Zarit burden score was highly correlated with the BAS score (correlation coefficient = 0.73, *p*<0.001) and the GHQ-28 total score (correlation coefficient = 0.62, *p*<0.001).(17) This questionnaire has been prepared by Navidian et al. (2008) based on the cultural conditions of Iran and its reliability has been estimated to be 0.94 through using retest method. In addition to content validity, its validity has been confirmed based on its positive and high correlation with Hamilton Anxiety Questionnaire (r = +0.89) and Beck Depression Inventory (r = +0.67).(18,19) The psychometric properties of the ZBI include an acceptable interitem reliability and convergent validity, indicated by a Cronbach’s alpha of 0.79 and a correlation coefficient of 0.71 between caregiver’s global evaluation and ZBI scores. Test-retest reliability (0.71) and internal consistency (Cronbach’s alpha= 0.91) have also been reported.(20-22) In the present study, the reliability of the ZBI was approved by Cronbach’s alpha=0.90.

### Procedures and statistical analysis

At first, the subjects were divided into intervention and control groups using random method. Before the intervention, the questionnaires were given to the samples. Then the intervention group subjects were educated. The training session was held by two researchers (a male researcher to teach male samples and a female researcher to train female samples) who had sufficient skills with regard to BRT. The educational content of the session included questions and answers about the benefits of relaxation and practical application of the technique. In this session, after explaining how to do the exercises, the caregivers were asked to perform the exercises in the presence of the researcher to ensure their accuracy.. After education, the caregivers performed the exercises independently. They did not receive special attention compared to the control group, in order to avoid more behavioral attention to the intervention group as a confounding factor as much as possible. In this study, patients and caregivers of the control group were not trained.

Benson's relaxation instructions are as follows: The caregivers are asked 1) To be in a comfortable position; 2) Close their eyes slowly; 3) Keep all the muscles of the body from the soles of the feet to the face gradually relax and keep calm; 4) Breathe through the nose and be aware of oneself and take the breath out slowly through the mouth and when the breath comes out, repeat the number one under the lips and breathe comfortably and normally; 5) Do this for 15 minutes and try to relax the muscles, then slowly open the eyes and do not get up for a few minutes; 6) Do not worry whether they have reached a deep level of calm or not, and let the relaxation happen by itself; when disturbing thoughts occur, try to ignore them and be indifferent to them.(23) As understanding happens better through watching, video tape was used to make Benson's relaxation instruction more intuitive. At the end, a copy of the CD together with an educational pamphlet was given to the intervention group. The caregivers of the patients were advised to perform this procedure twice a day for 15 minutes each time. It is important to do this procedure continually. 

The procedure was performed by the caregivers for one month. They were followed up in person and by telephone during this period. Necessary follow-up and reinforcement was also done in the form of SMS to ensure that the educational program was followed. The subjects of the control group did not receive any care. The ZBI questionnaire was completed in person again by both control and intervention groups one month after doing Benson's relaxation. The normality of the caregiver burden was checked using the Kolmogorov-Smirnov test. Normality was established.The accumulated data were analyzed by SPSS (version 16.0), using independent T-test, paired t-test, Ancova test, and chi-square. In this study, the significance level was considered at 0.05.

### Ethics approval and consent to participate

Ethical approval and permit of access to Mottahari hospital was obtained from Jahrom University of Medical Sciences. This study was approved by the Ethics Committee at Jahrom University of Medical Science (JUMS) with ethical code of [IR.JUMS.REC.1396.101]. The participants were informed and written consent was obtained before their participation in the study. Data confidentiality and anonymity was guaranteed for volunteers participating in the study. All methods were carried out by relevant guidelines and regulations. 

## Results

**Figure 1:**
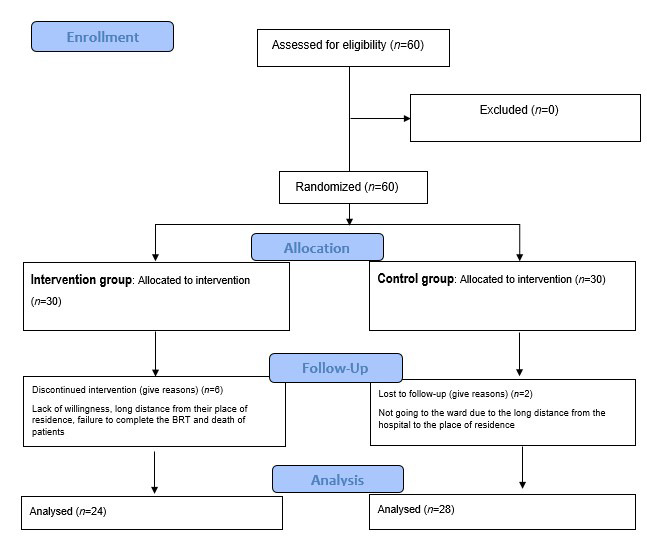
Flow chart of the study.

Fifty-two caregivers of the hemodialysis patients were divided into intervention (*n*=24) and control (*n*=28) groups (Figure 1). The majority of caregivers in the intervention 19 (79.2%) and control 18 (64.3%) groups were female. 17 patients in the intervention group (70.8%) and 26 in the control group most of the caregivers (92.9%) were married. However there was no significant difference between the groups in terms of demographic characteristics. It suggests that both groups are similar in terms of demographic characteristics (Table 1).

**Table 1:** Frequency of demographic variables in intervention and control group

Variables	Groups		*p*-value*
	Control group (*n*=28) Frequency (%)	Intervention group (*n*=24) Frequency (%)	
Sex			0.24
Female	18 (64.3)	19 (79.2)	
Male	10 (35.7)	5 (20.8)	
Age (years)			0.73
30 ≤	3 (10.7)	4 (16.7)	
40-31	7 (25.0)	8 (33.3)	
50-41	6 (21.4)	5 (20.8)	
50<	12 (42.9)	7 (29.2)	
Marital status			0.087
Single	2 (7.1)	5 (20.8)	
Married	26 (92.9)	17 (70.8)	
Widow	0 (0.0)	2 (8.3)	
Education			0.62
High school	13 (46.4)	10 (41.7)	
Diploma	10 (35.7)	7 (29.2)	
Above the diploma	5 (17.9)	7 (29.2)	
Occupation			0.62
Housewife	17 (60.7)	17 (70.8)	
Employed	7 (25.0)	6 (25.0)	
Retired	3 (10.7)	1 (4.2)	
Other	1 (3.6)	0 (0.0)	
Duration of care			0.79
1-30 days	6 (21.4)	8 (33.3)	
31-60 days	9 (32.1)	6 (25.0)	
61-90 days	2 (7.1)	3 (12.5)	
91-120 days	3 (10.7)	2 (8.3)	
More than 121 days	8 (28.6)	5 (20.8)	

*chi-square test

Based on the results of independent t-test before the intervention, there was no significant difference between the two groups in terms of the mean caregiver burden of caregiving (*p*>0.05).

After the intervention, the mean burden of providing care for hemodialysis patients in the caregivers of the intervention group was significantly lower than the control group (*p*<0.001). The results of paired t-test revealed that in the intervention group, the mean score of caregiver burden after the intervention (14.46±10.91) was significantly lower than before the intervention (38.33±16.94) (*p*=0.001) (Table 2).

**Table 2:** Comparison of mean caregiver burden in caregivers of hemodialysis patients before and after intervention in the control and intervention groups

	**Intervention group (*n*=24)** **Mean±SD**	**Control group (*n*=28)** **Mean±SD**	Independent t-test results*
Pre-intervention caregiver burden	38.33±16.94	35.75±19.33	T=-0.51 df= 50 *p*-value=0.61
Post-intervention caregiver burden	14.46±10.91	34.96±15.99	T=5.3 df= 50 *p*-value<0.001
Paired t-test results	T=6.002 df = 23 *p*-value=0.001^§^	T=-0.207 df = 27 *p*-value=0.84^§^	

*Independent t-test results; ^§^ Paired t-test

## Discussion

In general, the study showed that BRT reduces the caregiver burden in caregivers of hemodialysis patients compared to before the intervention. In this study, caregivers of hemodialysis patients who performed BRT reported less caregiver burden than control group. Another study showed that the caregiver burden was high in most caregivers of hemodialysis patients. 

Accordingly, given the high caregiver burden, caregivers of hemodialysis patients should have some programs to improve adaptation skills and control the factors affecting the increase of caregiver burden so that they can promote their health.(24) BRT reduces physical discomfort by reducing the activity of the automatic system. Moreover, it improves the ability to adapt and, by creating a feeling of relaxation in the muscles, leads to improved work, social and general activity adequacy and, ultimately, to a positive feeling in the person.(9) This includes mindfulness techniques which affect a wide range of physical and psychological signs and symptoms such as anxiety, pain, depression, mood and self-esteem and thus reduce stress.(25) 

 This study indicated that after Benson's relaxation, the mean and standard deviation of the caregiver burden score in the intervention and control groups were statistically significant and this method reduced the caregiver burden of the caregivers of the hemodialysis patients. In a study, the BRT was effective in the elderly hemodialysis patients, and reduced anxiety and depression, and improve sleep quality of these patients,(25) which is in line with our study. 

Another study similar to the present study in hemodialysis patients showed that BRT can be used as an alternative treatment to reduce depression, anxiety and stress and increase the quality of life of patients undergoing hemodialysis.(26) A study conducted in the emergency care showed that the patients undergoing BRT had a more significant decrease in anxiety score than others.(27) The similarity of the three mentioned studies with the present study can be due to the similarity in the type of intervention. Another reason for the similarity can be the type of variable studied in these studies (anxiety), which is somewhat similar to the type of variable reviewed in this study (caregiver burdens). The findings of a study provided insight into the problems of caregivers of patients undergoing hemodialysis. Patients with ESRD should have regular lifelong dialysis. Both the disease and its treatment (i.e. dialysis) have serious effects on patients and their caregivers. The long-term duties of caregivers in providing daily services to the patients can affect their social, financial and mental health. Initially, these caregivers are very eager to help these patients without receiving any salary; but over time, frustration and fatigue develop and cause serious social and psychological problems in them.(28) Another study showed that after Benson's relaxation, the mean and standard deviation of stress score were significantly different in the intervention and control groups. This study showed a significant reduction in stress after applying Benson's relaxation, while no significant changes were observed in the control group.(23) The results of this study were also in line with the results of the present study. The reason for the similarity of this study with the present study can be due to the similarity in the type of intervention and its effect on the type of variable mentioned (stress) which is similar to the type of variable evaluated in this study (caregiver burden). 

 In one study, the aim was to compare the effects of relaxation and aromatherapy inhalation on the fatigue of hemodialysis patients. In this clinical trial study, the subjects were classified into three groups of relaxation, aromatherapy and control. In the relaxation group, Benson's relaxation methods were used. In the aromatherapy group, the inhalation of two drops of 5% lavender essence was used and the control group received only regular care measures. The results of this study showed a significant difference in the mean of the changes in fatigue score before and after the intervention between the relaxation and aromatherapy groups, but this difference was not significant in the control group. In reducing the level of fatigue in hemodialysis patients, aromatherapy with lavender essence functioned better than BRT.(29) This finding is not in line with the findings of our study. 

The less effect of Benson's relaxation in this study than ours can be possibly due to the simultaneous use of aromatherapy and Benson's relaxation in this intervention. Moreover, this study has been conducted on hemodialysis patients and the study variable has been fatigue. By contrast, our study was conducted on the caregivers of hemodialysis patients and the variable of our study was caregiver burden. In a study that BRT was performed, the results showed that the level of anxiety, depression, well-being and work-related stress, and confidence to teach patients in nurses were not statistically significant. However, nurses reported more confidence in teaching this method to patients^.^(30) This may be due to the small sample size, which even the study itself suggests that larger studies may show a significant reduction in work-related stress and anxiety of nurses. Additionally, in order to reduce the level of job stress in nurses, other strategies should be employed as well. Another reason for the discrepancy was that the data collection tool in this study was different from our study.

The results of a similar study showed that BRT could be somewhat effective in reducing job stress of midwives.(31) In another study, research findings emphasized the significance of BRT as an alternative which can change oxidative stress markers, thereby reducing physical and mental symptoms.(32) Our findings of the study and other similar studies(8,23,27) show that the use of low-risk, low-cost, and convenience methods and performing them by the caregivers of hemodialysis patients can reduce the caregiver burden problems of these caregivers. These methods can be used in hemodialysis wards for the caregivers of hemodialysis patients as a routine in nursing care and beside the cares provided for the patients.

In the review article of Surani,(12) the eight articles with family-based intervention programs consisting of family-centered interventions, educational interventions, and psychological interventions showed significant results in reducing the burdens of caregivers who take care of hemodialysis patients. The results of this systematic review show that intervention programs could help reduce the burdens of family caregivers when caring for hemodialysis patients.

In a study conducted by Qane, (33) the results showed the effectiveness of supportive educative program on the burden on family caregivers of hemodialysis patients. Since the level of caregiver burden in caregivers of hemodialysis patients is high and these pressures can reduce the level of care for these patients and also endanger the caregiver's physical and mental health. As a result, they need nursing interventions, counseling and follow-up over time. Therefore, in hemodialysis patients, nurses should pay attention to the role of caregivers in the treatment of these patients, and interventions such as education of the patient and his caregiver, counseling, family therapy, and referral to support groups should be considered to reduce caregiving burden, so as to improve the quality of patient care and the physical and mental health of caregivers as hidden patients should be guaranteed, which will reduce the workload of nurses and increase the quality of care for hemodialysis patients. Nursing care needs to include both patients and their caregivers and support them. It is hoped that this study will guide nursing care in this direction.

### Limitations and strengths

As the strength of this study, it is one of the few studies that assessed the effect of Benson's relaxation technique on caregiver burden in caregivers of hemodialysis patients. Due to the positive effect of this method can be recommended as a complementary method of caregiver burden reduction. One of the limitations of this study was that the participants were selected from a single center, so the results cannot be generalized to other regions of the country. Another study limitation was that the procedure was performed only in caregivers of hemodialysis patients. It is suggested to be performed in caregivers of other chronic diseases.

## Conclusion 

 Based on the results of this study, BRT can have significant positive effects on reducing the caregiver burden of the caregivers of hemodialysis patients. Caregivers of hemodialysis patients play an important role in providing for these patients and the use of complementary medicine methods such as Benson's relaxation can have positive effects on these people without any complication. Moreover, using these exercises is both cost-effective and simple. Therefore, it is recommended that this method be considered in order to improve the level of psychological health and prevent increased caregiver burden, especially in caregivers of hemodialysis patients and other chronic diseases.
